# Induction of apoptosis and the regulation of ErbB signaling by laminarin in HT-29 human colon cancer cells

**DOI:** 10.3892/ijmm.2013.1409

**Published:** 2013-06-05

**Authors:** HEE-KYOUNG PARK, IN-HYE KIM, JOONGKYUN KIM, TAEK-JEONG NAM

**Affiliations:** 1 Department of Food and Life Science, Pukyong National University, Nam-gu, Busan 608-737, Republic of Korea; 2 Department of Biotechnology, Pukyong National University, Nam-gu, Busan 608-737, Republic of Korea

**Keywords:** laminarin, cell cycle, ErbB signaling pathway

## Abstract

Laminarin, found in marine brown algae, is used as a carbohydrate reserve for phytoplankton; however, it is also used in traditional Chinese medicine, and has been shown to have several biological activities, including anticancer activities. In this study, we examined the mechanisms through which laminarin from *Laminaria digitata* induces apoptosis in HT-29 colon cancer cells, as well as the involvement of the ErbB signaling pathway. Cell viability assay revealed that laminarin induced cell death in a dose-dependent manner. Cell cycle analysis revealed that laminarin increased the percentage of cells in the sub-G1 and G2-M phase. Western blot analysis demonstrated that laminarin inhibited the heregulin-stimulated phosphorylation of ErbB2. A decrease in cellular proliferation was also observed; this was found to be dependent on ErbB, which activates c-Jun N-terminal kinase. These findings demonstrate the important role of the epidermal growth factor receptor in colon cancer tumorigenesis, and suggest the potential of laminarin as a bio-functional food with anticancer effects on human colon cancer.

## Introduction

Recently, it was discovered that seaweed is composed of certain bioactive compounds, which have antitumor effects. Seaweed is also used in various functional materials and agents. In particular, seaweed polysaccharides, which comprise some of its bioactive compounds, include cellulose and viscous polysaccharides. Seaweed is also comprised of approximately 30–60% soluble polysaccharides; these polysaccharides include alginate, laminarin and fucoidan. Of these, laminarin is composed of β1–3 and β1–6-glucan, and is a storage glucan for brown seaweed (1). Due to these structural characteristics, laminarin has bioactivities similar to those of β-glucan; it has been shown to have immune-enhancing and anticancer effects, as well as antibacterial activity (2).

In this study, we investigated whether laminarin has direct anticancer activities in addition to its ability to inhibit cancer cell proliferation. We demonstrate that laminarin induces apoptosis in highly proliferative cancer cells. In a previous study, we showed that activated insulin-like growth factor receptor (IGF-IR) inhibits apoptosis (3). We showed that laminarin induces the apoptosis of HT-29 cells through the Fas and IGF-IR signaling pathways. Many human tumors express high levels of growth factors and their corresponding receptors, which contributes to cancer progression (4,5). Two such growth factors that induce cancer progression are IGF and epidermal growth factor (6).

In this study, we demonstrate that laminarin inhibits the activation of the ErbB pathway. The four members of the ErbB subfamily share a similar structure but have different functions (7,8). The overexpression of ErbB genes, particularly ErbB2, has been observed in human cancer (9). Heregulin (HRG) is co-expressed with ErbB2 proteins in human cancer cells, and heterodimerization with ErbB3 activates ErbB2 through an autocrine mechanism in colon cancer cells (10). Therefore, the HRG/ErbB2/ErbB3 pathway is an important regulator of aberrant growth in colon cancer (11,12). In our study, we confirmed that laminarin inhibits the proliferation and survival of colon cancer cells by regulating the ErbB receptor signaling pathway. Our results suggest that laminarin is a ligand of the high-affinity ErbB and IGF-1 receptors, and thereby significantly affects signaling by the two growth factors. Moreover, our results strongly suggest that in addition to inhibiting protein expression, specific mechanisms, such as apoptosis are involved in laminarin-associated cancer prevention.

## Materials and methods

### Cell culture

We used HT-29 colon cancer cells (ATCC HTB-38; ATCC, Manassas, VA, USA) to examine the effects of laminarin. The cells were maintained in a humidified environment comprised of 5% CO_2_ and 95% air at 37°C in RPMI-1640 supplemented with 10% fetal bovine serum (FBS), penicillin/streptomycin (Gibco BRL, Grand Island, NY, USA). The medium was changed every 2–3 days.

### Western blot analysis

To prepare a whole-cell extract, the cells were washed in PBS and suspended in extraction buffer [20 mM Tris (pH 7.5), 150 mM NaCl, 1 mM EDTA, 1 mM EGTA, 2.5 mM sodium pyrophosphate, 1 mM β-glycerophosphate, 1 mM Na_3_VO_4_, 1 μg/ml leupeptin, 1 mM PMSF and 1% Triton X-100]. Subsequently, 50 μg of boiling sample buffer were added to the total cell lysate, and the samples were boiled for 10 min at 100°C. Proteins in the extracts were separated by 7.5–15% SDS-PAGE and transferred onto polyvinylidene fluoride membranes (Millipore, Billerica, MA, USA). The membranes were blocked for 1 h at room temperature in blocking buffer [1% bovine serum albumin (BSA) in TBS-T] then probed with primary antibodies (1:1,000 in 1% BSA/TBS-T) overnight at 4°C. The membranes were then washed twice for 15 min each in TBS-T and incubated with peroxidase-conjugated goat anti-mouse or -rabbit antibodies (1:10,000 in 1% BSA/TBS-T).

### Cell cycle analysis

The cells were cultured in 6-well plates to 60% confluency then treated with serum-free medium (SFM) for 6 h followed by various doses of laminarin (0, 1.25, 2.5 and 5 mg/ml) for 24 h. The cells were then trypsinized, washed with PBS and treated with 50 μg/ml cold propidium iodide solution containing 0.1 mg/ml RNase A in PBS (pH 7.4) for 30 min in the dark. Flow cytometric analysis was performed on a FACSCalibur instrument (Becton-Dickinson, San Jose, CA, USA).

### Immunoprecipitation and western blot analysis

The cells were incubated in SFM for 24 h and stimulated with 100 ng/ml HRG. To prepare a whole-cell extract, cells were washed in PBS and suspended in extraction buffer [20 mM HEPES (pH 7.5), 150 mM NaCl, 1 mM EDTA, 1 mM EGTA, 100 mM NaF, 10 mM sodium pyrophosphate, 1 mM Na_3_VO_4_, 20 μg/ml aprotinin, 10 μg/ml antipain, 10 μg/ml leupeptin, 80 μg/ml benzamidine HCl, 0.2 mM PMSF and 1% Triton X-100]. For immunoprecipitation, cell lysates (750 μg) were incubated at 10°C with anti-ErbB2 antibodies. After 12 h, protein A-Sepharose beads were added to the cell lysates. The beads were collected by centrifugation for 2 min at 10,000 × g and washed 3 times with lysis buffer. The beads were then boiled with the immunocomplex in 1X sample buffer. The eluted proteins were analyzed by SDS-PAGE and western blot analysis.

### Statistical analysis

All variables were compared with an analysis of variance using SPSS software version 10.0 (SPSS Inc., Chicago, IL, USA). All values are presented as the means ± SD. A P-value <0.05 was considered to indicate a statistically significant difference.

## Results

### Laminarin induces a loss of mitochondrial membrane potential

The mitochondrial pathway is a critical apoptotic pathway that involves signaling by Bcl-2 family proteins. The mitochondrial pathway of apoptosis also involves changes in mitochondrial potential and the mitochondrial release of cytochrome *c* into the cytosol.

**Figure 1:**
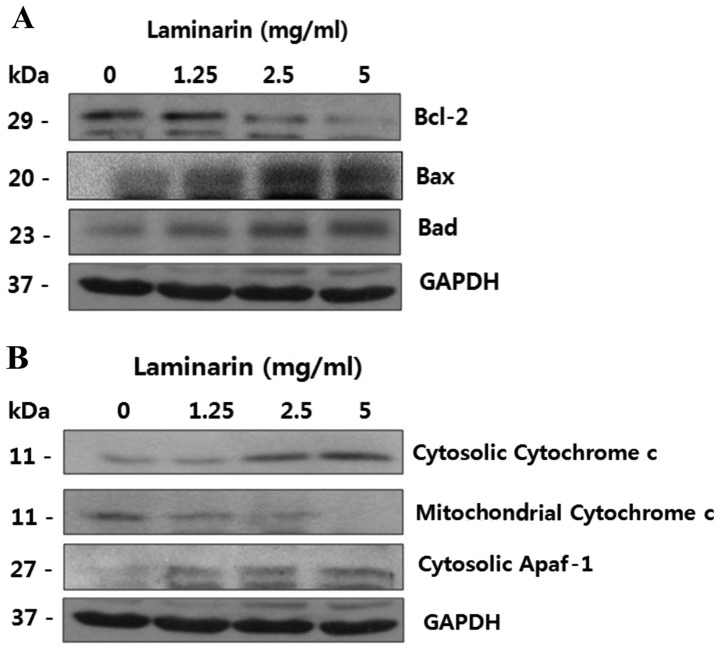
(A) Effects of laminarin on the expression levels of Bcl-2, Bax and Bad in HT-29 cells. Western blot analysis of Bcl-2, Bax and Bad protein expression. The cells were treated with laminarin (0, 1.25, 2.5 and 5 mg/ml). (B) Effects of treatment with laminarin on cytosolic and mitochondrial cytochrome *c* and apoptotic protease activating factor-1 (Apaf-1) expression in HT-29 cells. Laminarin-induced cytochrome *c* and Apaf-1 protein expression in the cytosol and mitochondria was examined by western blot analysis. The cytosolic levels of cytochrome *c* and Apf-1 increased, whereas mitochondrial cytochrome *c* protein levels decreased.

Thus, we monitored the expression of Bcl-2 family proteins. To determine whether laminarin triggers the release of cytochrome *c*, we examined the cytosolic and mitochondrial levels of cytochrome *c*. As shown in Fig. 1A, Bcl-2 expression decreased following treatment with laminarin, whereas Bad and Bax expression increased. Simultaneously, the levels of cytochrome *c* in the mitochondrial fraction decreased, whereas the levels in the cytosolic fraction increased and the expression of cytosolic apoptotic protease activating factor-1 (Apaf-1) also increased (Fig. 1B). This suggests a role for the mitochondria in laminarin-induced apoptosis.

### Effect of laminarin on cell cycle progression

**Figure 2:**
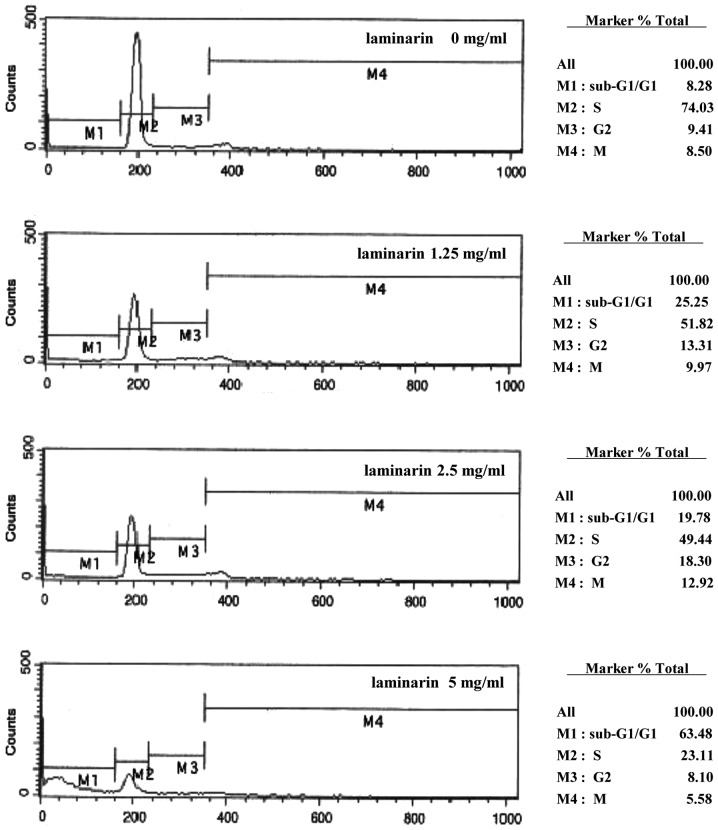
Laminarin-induced sub-G1 cell cycle arrest. DNA fluorescence histogram of HT-29 cell nuclei following treatment with laminarin (0–5 mg/ml) for 24 h, and the cell cycle was then analyzed by flow cytometry. Cell cycle analysis revealed that laminarin induced sub-G1 phase arrest in a dose-dependent manner.

Laminarin-induced apoptosis was assessed by cell cycle analysis (Fig. 2). The cell cycle response was examined in the cells treated with various concentrations of laminarin. We observed an increase in the percentage of cells in the sub-G1 and G2-M phase, while the percentage of cells in the other phases decreased. Treatment with laminarin markedly increased the proportion of cells in the sub-G1 and G2-M phase, suggesting that laminarin interferes with cell cycle progression.

### Effect of laminarin on the expression of cell cycle-related proteins

**Figure 3:**
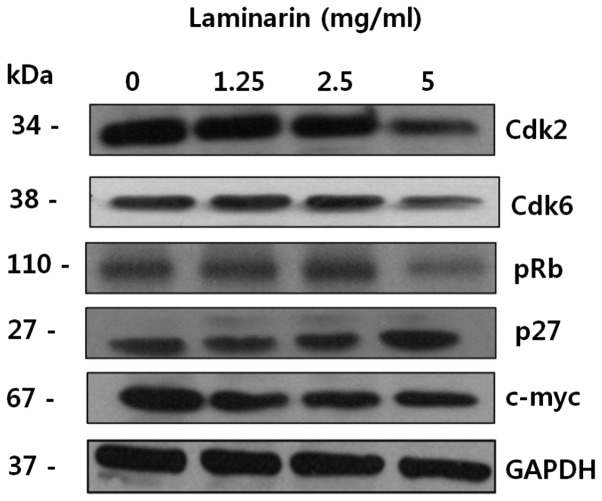
Effects of laminarin on the levels of cell cycle-related proteins in HT-29 cells. HT-29 cells were treated with laminarin (0–5 mg/ml) for 24 h. Cells were lysed and then total proteins were separated by SDS-PAGE. Proteins were visualized by western blot analysis using antibodies against Cdk2, Cdk6, pRb, p21 and c-myc.

To investigate the apoptotic mechanisms through which laminarin interferes with cell cycle progression, we confirmed the cell cycle-related protein content. The levels of p27, c-myc, pRb, Cdk2 and Cdk6 were measured by western blot analysis using specific antibodies against these proteins. The HT-29 cell cycle response was examined following treatment with laminarin at various concentrations. As shown in Fig. 3, the levels of Cdk2, Cdk6, pRb and c-myc decreased, whereas the p27 level in the nuclear fraction increased.

### Effect of laminarin on the expression of ErbB signaling pathway-related proteins

**Figure 4:**
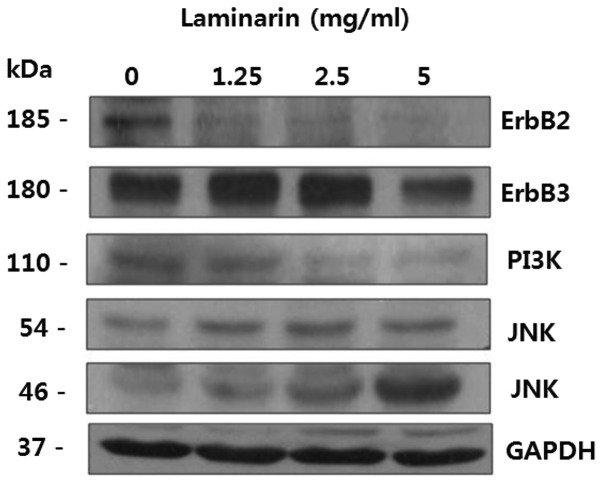
Effect of laminarin on ErbB2, ErbB3, PI3K, JNK and GAPDH expression. HT-29 cells were treated with laminarin (0–5 mg/ml) for 24 h. Equal amounts (50 μg) of cell lysates were subjected to SDS-PAGE and analyzed by western blot analysis using antibodies against ErbB2, ErbB3, PI3K and JNK.

ErbB receptor pathway-related proteins play important roles in normal cells, as well as in cancer cells. ErbB receptors control key pathways that govern cellular processes, such as proliferation, metabolism and survival (13,14). In tumor cells, ErbB2 activates ErbB3, which stimulates several intracellular signaling proteins and pathways, including MAPK, PI3K/Akt and Src kinase (13,15,16). As shown in Fig. 4, ErbB2, ErbB3 and PI3K expression decreased following treatment with laminarin, whereas that of JNK increased. These results suggest that laminarin alters the expression of ErbB signaling pathway-related proteins.

### Inhibition of HRG-induced p-Akt activation and ErbB2 phosphorylation by laminarin

**Figure 5:**
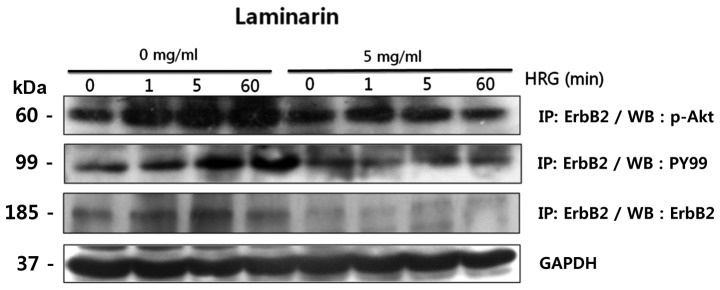
Effects of laminarin on HRG-induced p-Akt and PY99 expression. The HT-29 cells were treated with laminarin (5 mg/ml) for 24 h. HRG (100 ng/ml) was added 0–60 min prior to lysate (750 μg) preparation. Cell lysates were incubated with anti-ErbB2 antibodies and protein A-Sepharose beads to immunoprecipitate the HRG complexes. Total lysates were analyzed by western blot analysis for p-Akt, phospho-tyrosine and ErbB2.

The expression of p-Akt in the HT-29 cells treated with increasing levels of laminarin was examined by western blot analysis. As shown in Fig. 5, the recruitment of p-Akt, ErbB2 and PY99 was observed, which lasted 60 min in the control group. By contrast, protein expression was inhibited for up to 60 min following treatment with laminarin. The effect of laminarin on the HRG-induced association of ErbB2 and p-Akt was examined by immunoprecipitation using anti-ErbB antibodies followed by western blot analysis using anti-p-Akt antibodies. Additionally, we found that laminarin inhibited the HRG-induced increase in ErbB2 phosphorylation in HT-29 cells.

## Discussion

We have previously shown that treatment with laminarin inhibits the proliferation of colon cancer cells through the Fas and IGF-IR signaling pathways (3). In this study, we demonstrate that laminarin inhibits HT-29 cell growth through the intrinsic apoptotic and ErbB pathways.

In our previous study, we showed that laminarin induces apoptosis (3). Therefore, in this study, we examined the effects of laminarin on other apoptotic pathways. To our knowledge, this study provides the first evidence that laminarin decreases Bcl-2 family protein expression and inhibits cell cycle progression by regulating the ErbB signaling pathway.

Bcl-2 family proteins regulate apoptosis and the release of pro-apoptotic factors (17–19). Bcl-2 family protein and cytochrome *c* expression in the cytosol and mitochondria was detected. As shown in Fig. 1, laminarin increased the expression of apoptotic molecules, such as Bax and Bad, which belong to the Bcl-2 family. By contrast, the expression of anti-apoptotic molecules, such as Bcl-2 was decreased following treatment with laminarin. The anti-apoptotic factors, Bcl-2 and Bcl-xL, function by heterodimerizing with multidomain Bax effectors.

A loss of mitochondrial membrane potential is associated with apoptosis following the release of cytochrome *c*(20). The induction of Bax is associated with the release of cytochrome c from the mitochondria to the cytosol and the cleavage of poly(ADP-ribose) polymerase (21). As indicated in Fig. 1B, the release of cytochrome *c* from the mitochondria to the cytosol was induced following treatment with laminarin.

Apaf-1 plays a role in the activation of apoptosis in the intrinsic mitochondrial pathway. Apaf-1 induces the formation of apoptosomes and activates caspase-9. Activated caspase-9 then cleaves and activates downstream caspases, such as caspase-3, -6 and -7, leading to apoptosis (22). In this study, cytosolic Apaf-1 levels increased as the laminarin concentrations increased. As shown in Fig. 1, laminarin increased the expression of the apoptotic molecules, Bax and Bad, members of the Bcl-2 family, thus inducing apoptosis and mitochondrial dysfunction by the release of apoptotic factors, such as cytosolic cytochrome *c* and Apaf-1. Therefore, laminarin increased the protein levels of cytosolic cytochrome *c* and Apaf-1, suggesting that laminarin induced apoptosis through a mitochondrial-dependent pathway.

Laminarin-induced apoptosis was examined by cell cycle analysis (Fig. 2). The results revealed an increase in the percentage of cells in the sub-G1 and G2-M phase, while the percentage of cells in the other phases decreased following treatment with laminarin. Treatment with laminarin markedly increased the proportion of cells in the sub-G1-phase from 8.28 to 63.48%, those in the G2-phase from 9.41 to 18.30%, and those in the M-phase from 8.50 to 12.92%, suggesting that laminarin inhibits cell cycle progression.

As shown in Fig. 2, we found that laminarin induced a dose-dependent sub-G1 and G2-M phase cell cycle arrest; this was followed by apoptosis, which was associated with the expression of cell cycle-related proteins, such as Cdk and cyclin. The levels of Cdk2, Cdk6, pRb, p27 and c-myc were measured by western blot analysis. As shown in Fig. 3, the levels of Cdk2, Cdk6, pRb and c-myc decreased, whereas the level of p27 increased. Our results also indicated that laminarin-induced apoptosis was not associated with an alteration in p53 protein expression (data not shown). An increase in the levels of cyclin B1 and A regulates Cdk2 kinase expression at the G2-M phase (23). We demonstrated that laminarin induced cell cycle arrest, followed by cell death in highly proliferative colon cancer cells.

The dysregulation of the ErbB receptor family signal transduction pathway is observed in several types of cancer, including lung, breast, prostate, colon and duodenal cancer. The abnormal activation of the ErbB receptor family signal transduction pathway is considered one of the main causes of cancer (24).

ErbB family receptor tyrosine kinases are considered to play crucial roles in the incidence of cancer. These kinases include epidermal growth factor receptor (EGFR or ErbB1), ErbB2, ErbB3 and ErbB4. In addition, HRG is co-expressed with ErbB2 in colon cancer, and the autocrine activation of ErbB2 occurs through dimerization with ErbB3 (10). The extracellular domain of the ErbB receptor is responsible for ligand binding, inducing the formation of receptor dimers and the phosphorylation of tyrosine residues in the cytoplasmic domain of the receptor occurs through the activation of intrinsic tyrosine kinase. The phosphorylated tyrosine residues play a role in intracellular signaling. Phosphatidylinositol-3 kinase (PI3K) is activated through the binding of the SH domain in the p85 subunit to autophosphorylated tyrosine kinase receptors, and activated PI3K produces phosphatidylinositol-3,4,5-triphosphate, thus promoting the phosphorylation of Akt (25–27). Activated Akt is inactivated by the phosphorylation of the apoptosis-related protein; activated Akt is known to promote cell survival and suppress apoptosis (28).

ErbB receptor pathway-related proteins play important roles in normal cells; ErbB receptors regulate cell proliferation, metabolism and survival (13,14). In tumor cells, ErbB2 activates ErbB3 by stimulating several intracellular signaling proteins, such as MAPK, PI3K/Akt and Src kinase (13,15,16). As shown in Fig. 4, ErbB2, ErbB3 and PI3K expression levels decreased following treatment with laminarin, whereas the JNK expression level increased. These results suggest that laminarin inhibits the expression of ErbB signaling pathway-related proteins.

The HT-29 cells were incubated in SFM with the addition of 5 mg/ml of laminarin for 24 h; the cells were then stimulated with 100 ng/ml of HRG for 0, 1, 5 and 60 min. The effect of laminarin on the HRG-induced association of ErbB2 and p-Akt was examined by immunoprecipitation. As HRG binds with ErbB2, immunoprecipitation was carried out by the addition of ErbB2 antibodies to the cell lysates. The immunoprecipitated cell lysates were analyzed by western blot analysis. As shown in Fig. 5, the recruitment of p-Akt, PY-99 and ErbB2 was observed, which lasted 60 min in the laminarin-free treatment group (control group). By contrast, the protein expression of p-Akt, PY-99 and ErbB2 was inhibited for up to 60 min following treatment with laminarin. The HRG-induced ErbB2 protein expression levels were examined following treatment of the HT-29 cells with 5 mg/ml laminarin. Treatment of the HT-29 cells with laminarin inhibited phosphorylation and ErbB2 expression, as well as the phosphorylation of Akt; therefore, these results suggest that treatment with laminarin inhibits the proliferation of HT-29 cells.

In this study, we demonstrate that laminarin induces apoptosis through an apoptotic pathway involving growth factors and also demonstrate the effects of laminarin on the ErbB signaling pathway in HT-29 colon cancer cells. These findings suggest the important role of EGFR in colon cancer tumorigenesis, as well as the potential value of laminarin as a bio-functional food with anticancer effects on human colon cancer.

## References

[1] Painter TJ, Aspinall GO (1983). Algal polysaccharides. The Polysaccharides.

[2] Zvyagintseva TN, Shevchenko NM, Nazarova IV, Scobum AS, Luk’yanov PA, Elyakova LA (2000). Inhibition of complement activation by water-soluble polysaccharides of some far-eastern brown seaweeds. Comp Biochem Physiol C Toxicol Pharmacol.

[3] Park HK, Kim IH, Kim J, Nam TJ (2012). Induction of apoptosis by laminarin, regulating the insulin-like growth factor-IR signaling pathways in HT-29 human colon cells. Int J Mol Med.

[4] Kumar CC (1998). Signaling by integrin receptors. Oncogene.

[5] Hung MC, Lau YK (1999). Basic science of HER-2/neu: a review. Semin Oncol.

[6] Fürstenberger G, Senn HJ (2002). Insulin-like growth factors and cancer. Lancet Oncol.

[7] Peles E, Yarden Y (1993). Neu and its ligands: from an oncogene to neural factors. Bioessays.

[8] Carraway KL, Cantley LC (1994). A neu acquaintance for erbB3 and erbB4: a role for receptor heterodimerization in growth signaling. Cell.

[9] Hamdy FC, Thomas BG (2001). New therapeutic concepts in prostate cancer. BJU Int.

[10] Venkateswarlu S, Dawson DM, St Clair P, Gupta A, Willson JK, Brattain MG (2002). Autocrine heregulin generates growth factor independence and blocks apoptosis in colon cancer cells. Oncogene.

[11] Kapitanović S, Radosević S, Kapitanović M, Andelinović S, Ferencić Z, Tavassoli M, Primorać D, Sonicki Z, Spaventi S, Pavelic K, Spaventi R (1997). The expression of p185 (HER-2/neu) correlates with the stage of disease and survival in colorectal cancer. Gastroenterology.

[12] Safran H, Steinhoff M, Mangray S, Rathore R, King TC, Chai L, Berzein K, Moore T, Iannitti D, Reiss P, Pasquariello T, Akerman P, Quirk D, Mass R, Goldstein L, Tantravahi U (2001). Over expression of the HER-2/neu oncogene in pancreatic adenocarcinoma. Am J Clin Oncol.

[13] Hynes NE, Lane HA (2005). ERBB receptors and cancer: the complexity of targeted inhibitors. Nat Rev Cancer.

[14] Citri A, Yarden Y (2006). EGF-ERBB signalling: towards the systems level. Nat Rev Mol Cell Biol.

[15] Sharma SV, Settleman J (2009). ErbBs in lung cancer. Exp Cell Res.

[16] Yarden Y, Sliwkowski MX (2001). Untangling the ErbB signaling network. Nat Rev Mol Cell Biol.

[17] Reed JC (1997). Double identity for proteins of the Bcl-2 family. Nature.

[18] Chao DT, Korsmeyer SJ (1998). BCL-2 family: regulators of cell death. Annu Rev Immunol.

[19] Kluck RM, Bossy-Wetzel E, Green DR, Newmeyer DD (1997). The release of cytochrome c from mitochondria: a primary site for Bcl-2 regulation of apoptosis. Science.

[20] Shimizu S, Narita M, Tsujimoto Y (1999). Bcl-2 family proteins regulate the release of apoptogenic cytochrome c by the mitochondrial channel VDAC. Nature.

[21] Bossy-Wetzel E, Newmeyer DD, Green DR (1998). Mitochondrial cytochrome c release in apoptosis occurs upstream of DEVD-specific caspase activation and independently of mitochondrial transmembrane depolarization. EMBO J.

[22] Tang YJ, Yang JS, Lin CF, Shyu WC, Tsuzuki M, Lu CC, Chen YF, Lai KC (2009). *Houttuynia cordata* Thunb extract induces apoptosis through mitochondrial-dependent pathway in HT-29 human colon adenocarcinoma cells. Oncol Rep.

[23] Graña X, Reddy EP (1995). Cell cycle control in mammalian cells: role of cyclins, cyclin dependent kinases (CDKs), growth suppressor genes and cyclin-dependent kinase inhibitors (CKIs). Oncogene.

[24] Salomon DS, Brandt R, Ciardiello F, Normanno NP (1995). Epidermal growth factor-related peptides and their receptors in human malignancies. Crit Rev Oncol Hematol.

[25] Varticovski L, Harrison-Findik D, Keeler ML, Susa M (1994). Role of PI 3-kinase in mitogenesis. Biochim Biophys Acta.

[26] Toker A, Cantley LC (1997). Signalling through the lipid products of phosphoinositide-3-OH kinase. Nature.

[27] Klippel A, Kavanaugh WM, Pot D, Williams LT (1997). A specific product of phosphatidylinositol 3-kinase directly activates the protein kinase Akt through its pleckstrin homology domain. Mol Cell Biol.

[28] Datta SR, Brunet A, Greenberg ME (1999). Cellular survival: a play in three Akts. Genes Dev.

